# Adjuvant dendritic cell vaccination in high-risk uveal melanoma patients

**DOI:** 10.1186/2051-1426-3-S2-P127

**Published:** 2015-11-04

**Authors:** Kalijn Bol, Thomas van den Bosch, Gerty Schreibelt, Cornelis Punt, Carl Figdor, Dion Paridaens, Jolanda de Vries

**Affiliations:** 1 RIMLS, Radboudumc, Nijmegen, Netherlands; 2 Rotterdam Eye Hospital, Rotterdam, Netherlands; 3 Academic Medical Center, Amsterdam, Netherlands

## Background

The presence of monosomy 3 in the primary tumor is widely accepted as the most reliable prognostic parameter, identified in approximately 50% of patients with primary uveal melanoma [1]. Long term studies have shown a 3-year survival rate of 40% if monosomy 3 is present, whereas tumors with normal chromosome 3 status rarely give rise to metastatic disease and have a 90% 3-year survival rate [2]. Currently, there is no effective adjuvant treatment for the patients with early stage uveal melanoma at high risk for metastatic disease (monosomy 3). The development of uveal melanoma at an immune-privileged site, the eye, made it questionable if immunotherapy would be a suitable treatment modality. Nonetheless, in this open label Phase II study we investigated the immunological responses in high risk uveal melanoma patients, selected based on monosomy 3, to dendritic cell vaccination.

## Methods

Twenty-three patients with a primary uveal melanoma with monosomy 3 were included in this trial. HLA-A*02:01 positive patients received vaccinations with autologous dendritic cells loaded with melanoma antigens gp100 and tyrosinase. The main outcome measures are safety, immunological response, progression-free and overall survival. ClinicalTrials.gov Identifier: NCT00929019.

## Results

Tumor-specific immune responses were induced with dendritic cell vaccination in the majority of patients. Patients with a tumor-specific immune response showed a significant longer median progression free and overall survival compared to patients without a detectable tumor-specific immune response after dendritic cell vaccination. No severe treatment-related toxicities (common toxicity criteria grade 3 or 4) were observed. Updated data will be presented.

## Conclusions

Dendritic cell vaccination is feasible and safe in high risk uveal melanoma patients. Despite development of uveal melanoma at an immune-privileged site, dendritic cell-based immunotherapy is potent to enhance the host's anti-tumor immunity against uveal melanoma and tumor-specific immune responses correlate with longer progression free and overall survival.
